# CD8 T-cell subsets: heterogeneity, functions, and therapeutic potential

**DOI:** 10.1038/s12276-023-01105-x

**Published:** 2023-11-01

**Authors:** Choong-Hyun Koh, Suyoung Lee, Minkyeong Kwak, Byung-Seok Kim, Yeonseok Chung

**Affiliations:** 1 Laboratory of Immune Regulation, Research Institute of Pharmaceutical Sciences, Seoul National University, Seoul, 08826 Republic of Korea; 2 BK21 Plus Program, College of Pharmacy, Seoul National University, Seoul, 08826 Republic of Korea; 3 Division of Life Sciences, College of Life Science and Bioengineering, Incheon National University, Incheon, 22012 Republic of Korea; 4 Wide River Institute of Immunology, Seoul National University, Hongcheon, Gangwon 25159 Republic of Korea

**Keywords:** Lymphocyte differentiation, Interleukins

## Abstract

CD8 T cells play crucial roles in immune surveillance and defense against infections and cancer. After encountering antigenic stimulation, naïve CD8 T cells differentiate and acquire effector functions, enabling them to eliminate infected or malignant cells. Traditionally, cytotoxic T cells, characterized by their ability to produce effector cytokines and release cytotoxic granules to directly kill target cells, have been recognized as the constituents of the predominant effector T-cell subset. However, emerging evidence suggests distinct subsets of effector CD8 T cells that each exhibit unique effector functions and therapeutic potential. This review highlights recent advancements in our understanding of CD8 T-cell subsets and the contributions of these cells to various disease pathologies. Understanding the diverse roles and functions of effector CD8 T-cell subsets is crucial to discern the complex dynamics of immune responses in different disease settings. Furthermore, the development of immunotherapeutic approaches that specifically target and regulate the function of distinct CD8 T-cell subsets holds great promise for precision medicine.

## Expanding family of CD8 T cell: novel avenues in immunotherapy

In contrast to earlier beliefs that CD8 T cells primarily differentiate into cytotoxic T cells, recent advances have unveiled the presence of diverse subsets within the effector CD8 T cell population. Among these subsets, Tc17 cells have been extensively studied within the context of skin inflammation and the tumor microenvironment. Tc9 cells have shown links to specific autoimmune conditions alongside notable antitumor effects. Tfc cells, recognized for their role in supporting humoral responses, have also been identified within tumor microenvironments. Furthermore, regulatory CD8 T subsets have emerged in conditions such as graft-versus-host disease and autoimmune disorders, suggesting their potential contributions to immune modulation. These recent discoveries provide profound insights into the intricate landscape of immune regulation, offering promising avenues to harness or target these novel subsets for therapeutic interventions.

## Introduction

Conventional T cell populations consist of CD4 T cells and CD8 T cells that recognize cognate antigenic peptides presented by MHC class II and I molecules, respectively. Typically, CD4 T cells orchestrate overall immune responses through interactions with professional antigen-presenting cells, whereas CD8 T cells exhibit direct cytotoxicity against target cells. CD8 T cells are derived from lymphoid progenitor cells in the bone marrow and undergo further maturation in the thymus. Thymic mature CD8 T cells, known as naïve CD8 T cells, are poised to be activated after encountering specific antigens. Recent studies have highlighted the phenotypic and functional diversity among naïve CD8 T cells, revealing their heterogeneity^1^. The cytotoxicity-inducing ability of CD8 T cells was initially discovered in the infectious disease context^2–6^. After infection, naïve CD8 T cells proliferate and differentiate into effector CD8 T cells, enabling them to efficiently eliminate infected cells and protect the host from severe infection. Following antigen clearance, a fraction of effector CD8 T cells differentiate into memory cells, which can immediately proliferate upon re-exposure to the antigen, ensuring a swift and robust immune response^7–10^. However, when CD8 T cells are subjected to persistent antigen stimulation, as seen in chronic viral infections or tumors, they become exhausted, impairing their responses to subsequent antigen stimulation^11–13^.

Extensive research has focused on discerning the cellular biology of CD8 T cells driven by their potent cytotoxicity-inducing capacities, which has significantly advanced the field of tumor immunotherapy. This progress has led to the development of numerous immunotherapeutic strategies that harness the potential of CD8 T cells. The discovery of the cytotoxic function of CD8 T cells has paved the way for innovative therapeutic approaches. One prominent approach is adoptive cell therapy, which involves the isolation of CD8 T cells from patients and their subsequent ex vivo expansion and activation before being reintroduced back into the patient^14,15^. Specifically, significant strides have been made in the field of chimeric antigen receptor (CAR)-T-cell therapy, which involves genetically engineered patient CD8 T cells that effectively recognize cancer antigens. To date, the FDA has approved and clinically utilized more than six types of CAR-T-cell therapies for the treatment of certain types of hematologic malignancies^16,17^. Additionally, immune checkpoint inhibitors have emerged as a groundbreaking immunotherapeutic strategy that unleashes the full potential of CD8 T cells in response to tumors. By blocking inhibitory receptors, such as programmed cell death protein-1 (PD-1) or cytotoxic T-lymphocyte-associated protein-4 (CTLA-4), inhibitors restore the activity of CD8 T cells and promote their antitumor responses. This approach has shown remarkable success in certain types of cancer and has revolutionized the field of cancer immunotherapy.

Although many studies have traditionally been focused on cytotoxic CD8 T cells, also known as Tc1 cells, which are characterized by their production of cytolytic cytokines such as IFN-γ, granzyme B, and TNF-α, recent investigations have revealed additional subsets of effector CD8 T cells, each with distinct characteristics that differ from those of Tc1 cells. The cells in these subsets demonstrate reduced cytotoxicity but produce a diverse array of cytokines and express transcription factors that are similar to those expressed in CD4 T-cell subsets, suggesting that their effects extend beyond directly induced cytotoxicity. These cells play a pivotal role in triggering cytokine-mediated effector functions and actively participate in immune regulation and tissue homeostasis maintenance. Furthermore, they may contribute to the modulation of immune responses, the orchestration of inflammatory processes, and the coordination of immune cell interactions.

## Cytotoxic CD8^+^ T cells (Tc1 cells)

As typical cytotoxic CD8^+^ T cells, Tc1 cells produce perforin, granzyme B, IFN-γ, and TNF-α, which enable them to eliminate tumor and infected cells. The activation of Tc1 cells is promoted by IL-12, which is produced by antigen-presenting cells exposed to pathogen-derived maturation-promoting stimuli. Several key transcription factors, such as STAT4, T-bet, and EOMES, contribute to the polarization of Tc1 cells^14,18^. Activated Tc1 cells have traditionally been thought to kill tumor or infected cells through mechanisms involving perforin-granzyme and Fas-FasL signaling. However, recent studies suggest that Tc1 cells kill target cells through additional cytotoxic pathways, including ferroptosis and pyroptosis^19^.

Tc1 cells constitute the most prevalent subset of tumor-infiltrating lymphocytes in multiple types of cancers, including lung cancers^20^, breast cancers^21^, and chronic lymphocytic leukemia^22^, and are associated with favorable prognoses^23–27^. Tumors exhibiting a high degree of infiltration by Tc1 cells that subsequently produced elevated levels of IFN-γ are referred to as “hot” tumors. These “hot” tumors exhibit a more favorable response to immunotherapies than “cold” tumors, which lack Tc1 cell infiltration^28,29^. The expression of CD29 is associated with the increased cytotoxic potential of Tc1 cells in melanoma patients, suggesting that CD29 is a novel marker for the cytotoxic potency of Tc1 cells^30^.

The significance of Tc1 cells has also been established in the viral disease contexts, including patients infected with measles virus, cytomegalovirus, hepatitis C virus, and human immunodeficiency virus (HIV)^31–34^. Following the clearance of infected cells, effector Tc1 cells can differentiate into memory cells^7^. Memory Tc1 cells retain the cytotoxic phenotype of effector Tc1 cells^35^ and readily produce IFN-γ upon reactivation^36^. Even in antigen-naive mice, a population of memory-like Tc1 cells, namely, CD44^hi^CD122^hi^CD8^+^ T cells, which are different from naïve T cells, can rapidly produce IFN-γ in response to TCR stimulation^37–39^. These cells exhibit a distinct epigenetic pattern on the *Ifng* promoter, suggesting that cytokine production may be regulated at the transcriptional level independent of previous antigen exposure-induced cell priming. Memory Tc1 cells are crucial for protection against cancer and infection^40,41^. In particular, stem cell-like memory T cells show superior antitumor immunity compared to effector T cells^40^. Moreover, memory Tc1 cells are relatively unaffected by the inhibitory effects of regulatory T cells (Tregs) in the tumor microenvironment^42^. Thus, the frequency of memory Tc1 cells has been proposed to be a potential predictive marker for responsiveness to ICI treatment^43–45^.

Persistent antigen stimulation induces a state of exhaustion in effector Tc1 cells, leading to impaired cytolytic molecule production^11^. A single-cell transcriptome analysis revealed TOX as a key exhaustion-promoting transcription factor in human cancer^46^. Exhausted Tc1 cells display upregulated protein expression of TOX, which upregulates the protein expression of inhibitory receptors such as PD-1, LAG3, 2B4, and CD39^47,48^. The degree of Tc1 cell exhaustion is associated with poor outcomes for cancer patients^49^. Genetic engineering approaches have been used to demonstrate that the overexpression of BATF and IRF4 negatively regulated the expression of TOX in CD8 T cells, resulting in the generation of potent antitumorigenic CD8 T cells within the tumor microenvironment^50^. Compared to terminally exhausted PD-1^hi^Tim3^+^TOX^+^ Tc1 cells, progenitor exhausted PD-1^int^CXCR5^+^TCF-1^+^ Tc1 cells exhibited better control of tumor growth^12,51,52^. In contrast to terminally exhausted Tc1 cells, progenitor exhausted Tc1 cells are responsive to ICI immunotherapy^12,51^. Therefore, study into ways to reinvigorate exhausted Tc1 cells is an important research direction, as these cells hold great promise for developing novel immunotherapeutic approaches to chronic infections, cancers, and other diseases associated with Tc1 cell dysfunction.

## Non-Tc1 cell CD8 T-cell subsets

**Fig. 1:**
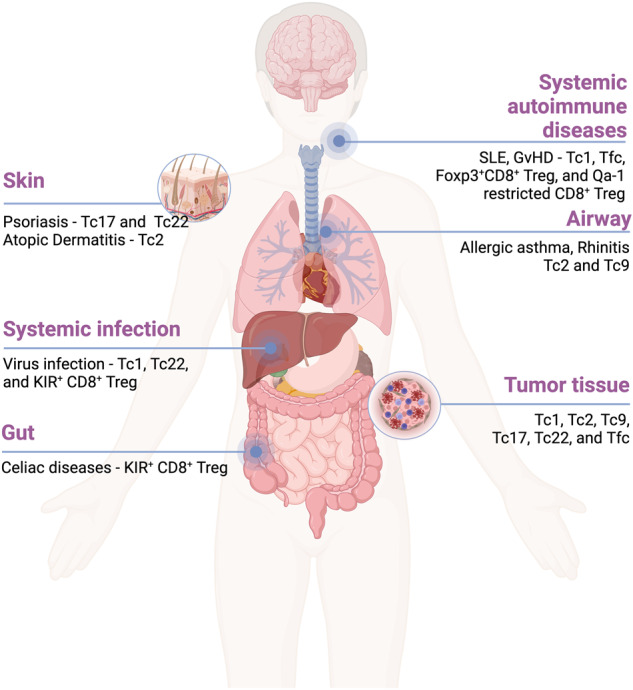
The roles of Tc subsets in diseases. Tc subsets play diverse roles in different diseases. Tc1 cells are known for their cytotoxic activity against tumors and infected cells, while Tc2 cells have been implicated in the pathogenesis of allergic diseases and cancer. Tc17 cells have been studied in the context of skin inflammation and the tumor microenvironment, while Tc9 cells have been associated with certain autoimmune disorders and antitumor effects. The role of Tc22 cells has been reported in psoriatic skin and tumor tissue. Tfcs support humoral responses in autoimmunity and are also found in the tumor microenvironment. Additionally, regulatory cell subsets such as CD8^+^ Foxp3^+^ Tregs, Qa1-restricted CD8^+^ Tregs and human KIR^+^ CD8^+^ Tregs have been identified in graft-versus-host disease and autoimmune diseases, in which they presumably contribute to immune regulation.

**Fig. 2:**
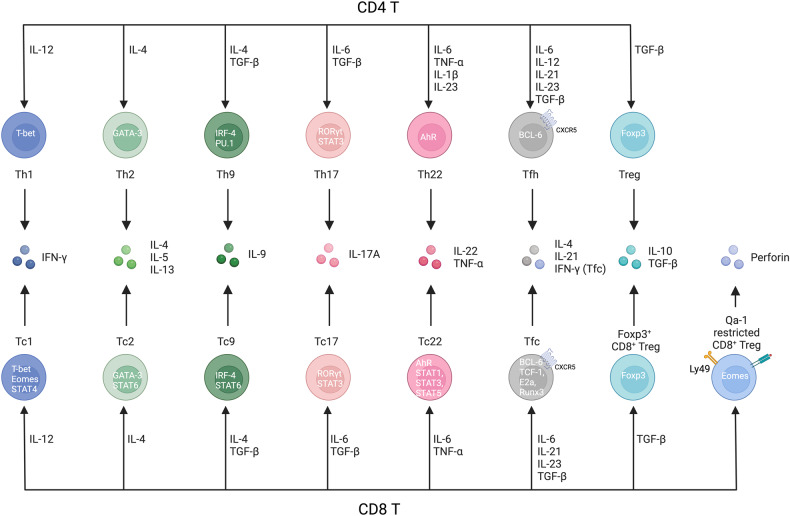
Comparison of Th cell and Tc subsets. Th cell subsets and Tc subsets share signal 3 cytokines, lineage-determining transcription factors, and effector cytokine profiles. Tc1 cells can be differentiated under IL-12 conditions and produce IFNγ through activation of the transcription factors T-bet, Eomes and STAT4. Tc2 cells can be differentiated under IL-4 conditions and produce IL-4, IL-5 and IL-13 through GATA-3 and STAT6. Tc9 cells can be differentiated under IL-4 plus TGF-β conditions and produce IL-9 through IRF-4 and STAT6. Tc17 cells can be differentiated under IL-6 plus TGF-β conditions through RORγt and STAT3. Tc22 cells can be differentiated under IL-6 plus TNF-ɑ conditions and produce IL-22 and TNF-ɑ through AhR, STAT1, STAT3 and STAT5. Tfcs can be differentiated under IL-6, IL-21, and IL-23 plus TGF-β conditions through BCL-6, TCF-1, E2A and Runx3. Foxp3^+^ CD8^+^ Tregs can be differentiated under TGF-β conditions and produce IL-10 and TGF-β through Foxp3. Qa-1-restricted CD8^+^ Tregs recognize the Qa-1 peptide on MHC class I molecules and suppress Qa-1-expressing T cells by secreting perforin meditated through the Eomes signaling pathway.

Although Tc1 cells represent the major population of CD8 T cells, alternative CD8 T-cell subsets have been identified in various diseases in both animal and human models (Fig. 1). CD8 T-cell subsets closely resemble their CD4 T-cell subset counterparts, as both CD4 T cells and CD8 T cells share similar signal 3 cytokine requirements, lineage-determining transcription factors, and effector cytokine profiles (Fig. 2, Table 1).

**Table 1:** Characterization of CD8 T-cell subsets.

Subset	Surface markers	Cytokine requirements	Effector cytokines	Transcription factors
Tc1 cells	CD49d	IL-2, IL-12	Perforin, granzyme B, IFN-γ, TNF-α	STAT4, T-bet, EOMES
Tc2 cells	CysLT1, BLT-1	IL-4	IL-4, IL-5, IL-13	STAT6, GATA3
Tc9 cells	IL-9R	IL-4, TGF-β	IL-9, IFN-γ	STAT6, IRF4
Tc17 cells	CD161, CD26, CD6, CD39, CD69, CD120b and PD-1	IL-6, TGF-β, IL-1β, IL-21, IL-23	IL-17, IL-22, GM-CSF	STAT3, RORγt
Tc22 cells	IL-6R	IL-6, IL-21, TNF-α	IL-22, IL-17	AhR, STAT1, STAT3, STAT5
Tfcs	CXCR5	IL-6, IL-21, IL-23, TGF-β	IL-4, IL-21, IFN-γ	TCF-1, BCL-6, E2a, Runx3
Qa1-restricted CD8^+^ Tregs	CD122, Ly49	IL-15	TGF-β, perforin	Eomes
Foxp3^+^ CD8^+^ Tregs	CD103	TGF-β	IL-10, TGF-β	FOXP3

### Tc2 cells

A subset of CD8 T cells producing Th2 cytokines, termed Tc2 cells, has been found in airway and intraepithelial tissues^53,54^. In vitro stimulation of naïve CD8 T cells with IL-4 induced IL-4- and IL-5-producing CD8 T cells^55,56^. Th2 cell cytokine production by Tc2 cells was promoted by the transcription factors STAT6 and GATA3, similar to effect of these transcription factors in Th2 cells^14^. Similar to Th2 cells, Tc2 cells stimulate B-cell IgE production, recruit eosinophils, and contribute to allergic responses^57,58^.

In the context of allergic asthma, the number of Tc2 cells is increased in severe eosinophilic asthma in humans^58^. Interestingly, Tc2 cells produce type 2 cytokines in response to stimulation by PGD2 and LTE4, which are major lipid mediators released by mast cells during an allergic response^58,59^. Compared to Th2 cells, Tc2 cells are less responsive to corticosteroid treatment, highlighting the potential of Tc2 cells as a therapeutic target for steroid-resistant asthma^57,60^. In steroid-resistant asthma, Cyp11a1, a mitochondrial enzyme that cleaves side chains, has been identified as a key regulator in the differentiation of Tc2 cells^61^. Moreover, the pathogenicity of Tc2 cells appears to be heightened in a hypoxic environment, as evidenced by the increased production of IL-13^62^. Similarly, in allergic rhinitis, CD8 T cells release IL-4 and contribute to the pathogenesis of disease^63^. After allergen-induced immunotherapy, the percentage of IL-4-producing CD8 T cells tends to be significantly reduced in patients with intermittent allergic rhinitis^64,65^.

Individuals with allergic dermatitis (AD), similar to those with asthma, present with a higher frequency of Tc2 cells^66,67^. In healthy individuals, Tc2 cells make up ~1% of CD8 T cells, but this proportion is increased to ~4% in AD patients^68^. Histamine, a potent inflammatory mediator, promotes cross-presentation of antigens by dendritic cells and thus induces the accumulation of Tc2 cells^69–71^. Single-cell RNA sequencing and proteomic analysis of AD patients who had been treated with the IL-4Ra-blocking antibody dupilumab revealed the presence of Tc2 cells in the skin that were not detected in healthy controls, indicating the persistence of tissue-resident memory Tc2 cells^72^.

Although effector memory Tc1 cells are capable of producing high levels of IFN-γ and cytotoxic granules, effector memory Tc2 cells lack this ability and are ineffective in killing target cells^73^, suggesting that skewing Tc1 cell transition into Tc2 cells can compromise the antitumor function of CD8 T cells. In cervical cancer patients, tumor cells promote the acquisition of a Tc2 cell phenotype by tumor-infiltrating CD8 T cells, which leads to increased production of IL-4 and decreased production of IFN-γ, facilitating immune escape of tumor cells^74^. Similarly, in urothelial bladder cancer, the exhaustion and reduced cytotoxicity of CD8 T cells in sentinel nodes are attributed to a decline in perforin expression caused by the Tc2 cell-polarized tumor microenvironment, which leads to an exhausted effector memory phenotype^75^.

### Tc9 cells

IL-9-producing CD8 T (Tc9) cells are transcriptionally regulated by STAT6 and IRF4, which are transcription factors of Tc2 and Th9 cells, respectively^14^. Stimulation of naïve CD8 T cells in the presence of IL-4 and TGF-β induces the differentiation of Tc9 cells in vitro^76^. Functionally, both Tc2 and Tc9 cells are implicated as pathogenic drivers of allergic conditions such as allergic asthma and atopic dermatitis^57,76^. Although not as extensively investigated as Tc2 cells, Tc9 cells are also linked to eosinophilia and elevated levels of FeNO (fractioned exhaled nitric oxide), a noninvasive marker of inflammation in asthma patients^77^. Although the transfer of Tc9 cells alone is insufficient to induce symptoms of asthma, their co-transfer with Th2 cells results in severe airway inflammation is characterized by an increased number of eosinophils in bronchoalveolar lavage (BAL) and an elevated lung inflammatory score^76^. In addition, the number of Tc9 cells is also increased in atopic dermatitis in both mice and humans^76^.

Importantly, Tc9 cells have been identified within the tumor tissue of breast cancer patients, and there is a positive correlation between the transcription levels of *IL9* and *IL9R*^78^. In contrast to that of Tc2 cells, the adoptive transfer of Tc9 cells led to potent antitumor effects in animal models^79^. A transcriptome analysis suggested that Tc9 cells in the tumor undergo transcriptional modifications related to cholesterol synthesis and efflux. Mechanistically, the activation of liver X receptor by oxidized cholesterol negatively regulated the differentiation and antitumor activity of Tc9 cells^80^. A recent study demonstrated that lipid peroxidation plays a crucial role in regulating the stability of Tc9 cells and their antitumor activity by regulating the IL-9-STAT3-fatty acid oxidation axis^81^. The emerging understanding of Tc9 cells as key players in allergic conditions, asthma pathogenesis and cancer immunity may open new avenues to therapeutic interventions.

### Tc17 cells

Tc17 cells are defined as CD8 T cells that produce IL-17 and express the transcription factors STAT3 and RORγt^82^. In different research contexts, there is ongoing debate among researchers regarding the characteristics of Tc17 cells. Some argue that Tc17 cells express T-bet, which is the master regulator in Th1 and Tc1 cells^83^. However, opposing arguments have suggested that Tc17 cells exhibit limited cytolytic activity and express minimal levels of granzyme B and perforin, thereby distinguishing them from Tc1 effector cells^84^. Depending on the disease status and bodily location, Tc17 cells can secrete other cytokines, such as IL-22, GM-CSF, IL-5, and IL-13^85,86^.

**Fig. 3:**
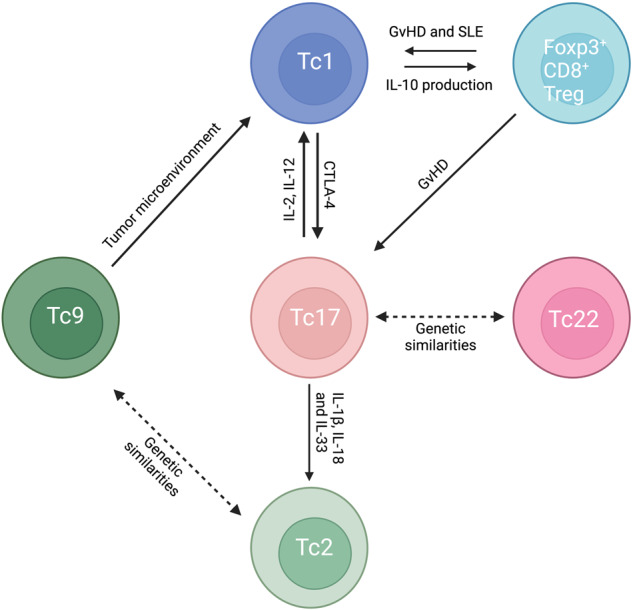
Plasticity among Tc subsets. Tc17 cells, akin to Th17 cells, display high plasticity in terms of cytokine production under specific conditions, and conversely, other Tc subsets can transform into Tc17 cells within certain contexts. Adoptive transfer of in vitro differentiated Tc17 cells led to their production of IFN-γ and granzyme B, similar to Tc1 cells. CTLA-4 plays a role in shifting the programming of Tc1 cells toward Tc17 cell differentiation. In the skin, alarmins such as IL-1, IL-18, and IL-33 stimulate commensal bacteria-specific Tc17 cells to produce Tc2 cytokines. Tc17 cells and Tc22 cells exhibit shared features, including similar differentiation-inducing cytokines, signaling transcription factors and cytokine production profiles. In a GvHD model, some adoptively transferred Foxp3^+^CD8^+^ Tregs were transformed into Tc17 and Tc1 cells. In addition to Tc17 cells, constituting another Tc subset also exhibit plasticity. Tc1 cells can produce IL-10 and exhibit suppressive functions in the context of viral infection. Tc2 and Tc9 cells share common features, including differentiation-inducing cytokines, signaling transcription factors and cytokine production profiles. In the tumor microenvironment, Tc9 cells undergo conversion to Tc1 cells, leading to potent antitumor immune responses, whereas Tc2 cells do not significantly contribute to attenuated tumor development.

The heterogeneity in cytokine profiles and transcription factors may be partly due to the high plasticity of Tc17 cells (Fig. 3). Multiple studies have confirmed the minimal cytolytic activity of Tc17 cells generated in vitro^87–89^. Interestingly, when in vitro-generated Tc17 cells were adoptively transferred in vivo, they lost IL-17 expression and acquired a Tc1-like phenotype and function; therefore, they secreted IFN-γ and granzyme B^89^. The activation of the PI3K/AKT pathway has been suggested to play a crucial role in the transdifferentiation of Tc17 cells into Tc1 cells, with Eomes upregulation being an essential component of this reprogramming process^90^. Furthermore, the commensal bacteria-specific response of Tc17 cells has been shown to drive these cells to acquire a Type 2 transcriptome that can be awakened after tissue injury and subsequent production of alarmins, such as IL-1, IL-18, IL-25, and IL-33^86^. On the other hand, the plasticity of Tc17 cells was negatively regulated by immune checkpoints, specifically PD-1 and CTLA-4^91,92^. The cross linking of CTLA-4 on Tc17 cells enhances their ability to maintain a Tc17 cell profile and resist transitioning into Tc1 cells, even under Tc1-transdifferentiation-skewing conditions^91^. Similarly, PD-1 signaling suppressed not only the differentiation of Tc17 cells but also the conversion of Tc17 cells into Tc1 cells, thereby reducing the cytotoxic potential of the overall Tc1 cell population^92^. The various signaling pathways involved in Tc17 polarization explain the heterogeneity and plasticity of Tc17 cells.

Tc17 cells induce the production of antimicrobial peptides and chemokines, which attract neutrophils to sites of infection or inflammation, thereby contributing to immune defense responses against pathogens such as bacteria and fungi^93,94^. However, an imbalance or overabundance of Tc17 cells can lead to the pathogenesis of autoimmune inflammatory diseases, including but not limited to Crohn’s disease and psoriasis. In Crohn’s disease, a chronic inflammatory bowel disease that primarily impacts the gastrointestinal tract, Tc17 cells, which normally contribute to gut health by aiding in repair and barrier function, demonstrate altered gene expression patterns associated with cytotoxicity and heightened production of the inflammatory cytokine TNF-α. As expected, patients with Crohn’s disease exhibit a higher frequency of Tc17 cells than healthy individuals^95^. Moreover, high-dimensional profiling studies identified enrichment of Tc17 cells in active Crohn’s disease samples, specifically highlighting a subset of cells expressing CD6^high^, CD39, CD69, PD-1, and CD27^low^, which may serve as targets for therapeutic interventions^96^.

In psoriasis, Tc17 cells are key contributors to disease onset, arguably playing a more prominent role than Th17 cells^85,97–99^. A markedly increased number of Tc17 cells are recruited to psoriatic lesions compared to the number in normal skin, whereas no significant difference has been found in Th17 cell levels. Moreover, the frequency of Tc17 cells in the peripheral blood of psoriasis patients correlated with disease severity^99^. A single-cell transcriptomic analysis identified distinct inflammatory Tc17 cell subsets enriched in psoriatic lesions that commonly express CXCL13, a biomarker of psoriasis severity^98^.

Memory Tc17 cells possess both tissue-resident and effector memory properties. Despite their high plasticity as effector cells, as antifungal vaccine-induced memory cells, Tc17 cells persisted in maintaining a stable type 17 phenotype and were not converted into Tc1 cells^100^. Most memory Tc17 cell with activation induced by an antifungal vaccination expressed CD103, an integrin and marker of tissue-resident memory cells, and high levels of CD127 (IL-7Ra); thus, sharing similar phenotypes with tumor-infiltrating Tc17 cells while also expressing phenotypic markers, namely, CD62L^lo^ and Ly6C^lo^, that are consistent with effector memory cells^87^.

**Fig. 4:**
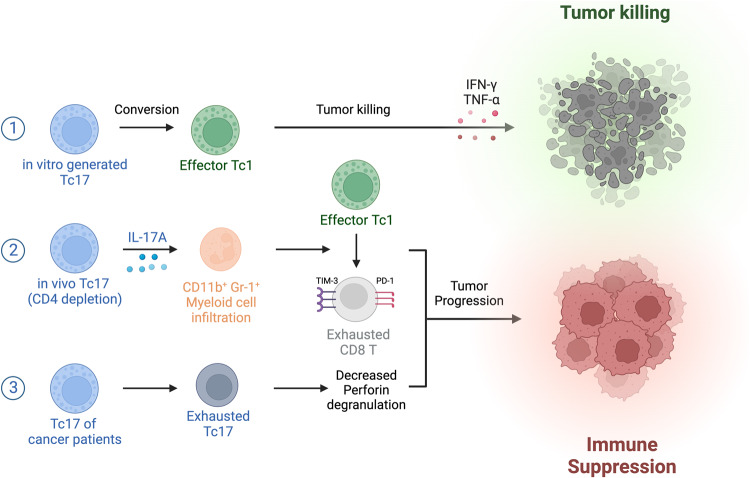
Controversial role of Tc17 cells in the tumor microenvironment. Tc17 cells in the tumor microenvironment play distinct roles in tumor growth depending on their origin. ① In vitro-generated Tc17 cells are converted to effector Tc1 cells and repress tumor cell growth. On the other hand, Tc17 cells generated in vivo have been shown to exhibit pro-tumorigenic effects. ② Tc17 cells induced in the tumor microenvironment followed by CD4 depletion to recruit infiltrating myeloid cells and accelerate the exhaustion of effector Tc1 cells. ③ Tc17 cells from cancer patients presented with a terminally exhausted phenotype, which resulted in impaired antitumor immune responses.

The roles of Tc17 cells in the tumor microenvironment is still debated (Fig. 4). Memory Tc17 cells, with enhanced self-renewal abilities and potential for long-lasting memory, are considered promising candidates for cancer immunotherapy^89^. In contrast to the stable Tc17 cells observed after fungal vaccination, in vitro-generated Tc17 cells were easily converted into Tc1 cells in tumor environments; therefore, they served as a reservoir of Tc1 cells in vivo^89^. On the other hand, in vivo-generated Tc17 cells exhibited a stable phenotype and have been shown to promote the exhaustion of Tc1 cells, presumably by recruiting CD11b^+^Gr-1^+^ MDSCs in animal models^87^. Additionally, a specific subset of Tc17 cells, characterized by low PD-1 expression and high OX40 expression, has been associated with reduced patient survival rates^83^. In hepatitis B virus infection, IL-17 attracted CD11b^+^Gr-1^+^ MDSCs to trigger CD8 T-cell exhaustion^101^, whereas in myelodysplastic syndromes, CD11b^+^Gr-1^+^ MDSCs have been proposed to induce CD8 T-cell exhaustion via the Tim-3/Galectin-9 pathway^102^.

### Tc22 cells

Tc22 cells, constituting a less-extensively investigated subgroup of CD8 T cells, are known for their production of IL-22. IL-22 belongs to the IL-10 family of cytokines and primarily targets epithelial cells, keratinocytes, hepatocytes, and pancreatic β cells^103^. Similar to IL-17, IL-22 maintains the epithelial barrier by promoting tissue repair and wound healing^104^. Tc22 cells share similarities with Tc17 cells, as they produce a small amount of the IL-17 cytokine, and Tc17 cells produce IL-22^14^. Moreover, the cytokine requirements for the in vitro differentiation of Tc22 cells resemble those of Tc17 cells; that is, they require the IL-6 and IL-21 cytokines^103,105^. In line with this, there have been reports of IL-17 and IL-22 being overrepresented in coproducing CD8 T cells in psoriatic lesions^85^, raising questions about whether Tc22 is truly a distinct T-cell line. However, further inspection of dermal samples revealed an increase in IL-22-producing CD8 T cells that lacked IL-17 expression in psoriatic lesions, reinforcing the idea that Tc22 cells constitute a separate T-cell subset^85^.

A recent investigation has shed light on the role of Tc22 cells in the context of tumors. In vitro-generated Tc22 cells exhibited high cytolytic activity and effective control of tumor growth when transferred into tumor-bearing hosts, and their effects were comparable to or even more pronounced than those of Tc1 cells^103^. Intriguingly, Tc22 cells have been detected in populations of tumor-infiltrating lymphocytes (TILs) from ovarian cancer tissue that had been expanded, thereby comprising up to ~35% of the CD8 T cells in some patients, and the production of IL-22 by these CD8^+^ TILs correlated with increased recurrence-free survival. This finding suggested that directing T cells toward differentiation into a Tc22 cell lineage may hold promise for advancing cell therapies such as CAR-T-cell or TCR-based immunotherapy^103^. In transplant-associated squamous cell carcinoma (TSCC), however, an increase in the number of Tc22 cells was associated with a decrease in the number of Th1 cells and an increase in tumor growth, suggesting that Tc22 cells may contribute to the progression of TSCC^106^.

In the context of viral infection, individuals exposed to HIV but not infected with the virus exhibited a higher frequency of Tc22 cells compared to their HIV-infected partners, who tended to produce a relatively higher proportion of Tc17 cells, suggesting the protective role of Tc22 cells against viral infections such as HIV^107^. In the acute phase of SARS-CoV-2 infection, which causes COVID-19, an observed increase in the frequency of Tc22 cells compared to that in healthy control groups has been observed. Tc22 cells have been associated with milder symptoms or even asymptomatic cases, suggesting a potential protective effect against SARS-CoV-2 infection^108^.

### Follicular cytotoxic T cells (Tfcs)

Follicular cytotoxic T cells (Tfcs) are a recently explored subset of CD8 T cells. Similar to follicular helper T cells (Tfh cells), Tfc cells express the chemokine receptor CXCR5, which facilitates their migration to the germinal center. The differentiation of Tfc cells is induced by the cytokines IL-6, IL-21, IL-23, and TGF-β, which activate multiple transcription factors, including TCF-1, BCL-6, E2a, and Runx3, playing important roles in the differentiation of Tfc cells^109,110^. Extensive research has been conducted to investigate the role of Tfc cells in both the humoral response and the antitumor response.

Tfc cells were initially discovered in the B-cell follicle of human tonsils^111^. When cocultured with B cells, Tfc cells enhance both the survival and antibody production of B cells^111^. IL-2 knockout mice, which lack regulatory T cells and are susceptible to autoimmune diseases, exhibited a notable increase in the population of Tfc cells. Notably, depletion of CD8 T cells led to a decrease in B-cell frequency and autoantibody production and an increase in survival rate. These findings indicate that Tfc cells play a crucial role in enhancing B-cell responses in vivo^112^. Tfc cells not only function as B-cell helpers themselves but also synergistically cooperate with Tfh cells to facilitate B-cell maturation and antibody class switching^112,113^. Mechanistically, Tfc cells support B-cell function through the secretion of IL-21 and CD40L^113^. The increased antibody production by Tfc cells in human and mouse models has prompted clinical investigations into the correlation between Tfc cell frequency and the severity of viral infections. In HIV infection, it has been reported that the quantity of Tfc cells increases in HIV-infected patients compared to healthy controls, and their frequency is inversely correlated with the viral load of HIV^114–116^. In contrast to the B-cell helper function of Tfc cells, recent studies have revealed a distinct population of IFN-ɣ^+^CXCR5^+^ Tfc cells that exert suppressive effects on antibody production in both mouse and human models^117,118^. In the transplantation model, the presence of IFN-ɣ^+^CXCR5^+^ Tfc cells inhibits alloantibody production^117^. The suppressive function of these cells is dependent on IFN-ɣ, as demonstrated by the inability of IFN-ɣ-deficient CD8 T cells to suppress antibody production^119^.

In addition to their role in regulating antibody responses, the role of Tfc cells has been reported in several cancer studies. Tfc cells are found in a wide range of tumors, including both solid tumors, such as non-small cell lung cancer, and liquid tumors, such as follicular B-cell non-Hodgkin’s lymphoma^120–122^. Tfc cells in the tumor microenvironment are characterized by their expression of TCF1 and PD-1 and low levels of TIM3. These cells exhibit a less cytotoxic phenotype than effector cells and share similarities with progenitor exhausted Tc1 cells, displaying limited proliferation and cytokine production^123,124^. Additionally, Tfc cells possess self-renewal capacity and responsiveness to immune checkpoint blockade, which are characteristics of progenitor exhausted Tc1 cells^125,126^. These progenitor exhausted phenotypes of Tfc cells suggest their potential as promising therapeutic targets in cancer treatment. Clinical studies have revealed a correlation between the frequency of Tfc cells and disease-free or overall survival in various cancers, including pancreatic, colon, follicular lymphoma, gastric, high-grade serous ovarian, hepatocellular, and bladder cancers^127–132^. Collectively, Tfc cells are emerging as a potential biomarker for predicting prognosis in both viral infections and cancer.

### CD8^+^ Tregs

Regulatory T cells are crucial for maintaining immune homeostasis and preventing autoimmune disorders. Although CD4^+^ Foxp3^+^ cells are recognized as predominant regulatory T cells, multiple studies have demonstrated that CD8^+^ Tregs play immune-suppressing roles in diverse human and murine systems^133–135^.

The suppressive functions of CD8 T cells, restricted to the nonclassical MHC class I molecule Qa-1, in humoral responses was discovered in β_2_m-knockout mice^134,136^. These suppressive CD8^+^ Tregs recognize the Qa-1 peptide and are characterized by their expression of CD122 and Ly49 molecules. The suppressive activity of Qa-1-restricted CD8^+^ Tregs depends on the IL-15 signaling pathway^137,138^. Qa-1-restricted CD8^+^ Tregs are essential for self-tolerance mediated through the inhibition of Qa1^+^ follicular helper T cells, and Helios is required for the terminal differentiation of these cells^138–140^. Recent studies have provided evidence highlighting the essential roles of TGF-β and Eomes in maintaining the homeostasis of Qa-1-restricted CD8^+^ Tregs, each with distinct contributions. TGF-β signaling maintains the stability of Qa-1-restricted CD8^+^ Tregs by upregulating Helios expression, and the genetic program governed by Eomes directs the localization of these cells to the germinal center^141^. Along with lupus-like autoimmune disease, Qa-1-restricted CD8 Tregs directly regulated autoreactive CD4 T cells in an experimental autoimmune encephalomyelitis (EAE) model^142–146^. Specifically, Qa-1-deficient CD4 T cells escaped the suppressive effects of Qa-1-restricted CD8 Tregs and developed EAE^142^.

Suppressive Qa-1-restricted CD8^+^ Tregs have also been identified in humans, and their frequency is highly increased in patients with autoimmune diseases as well as those infected with SARS-CoV-2virus^147–149^. Suppressive human CD8^+^ Tregs express killer immunoglobulin-like receptors (KIRs), which are the evolutionary equivalent to Ly49 receptors in mice. Patients with autoimmune diseases present with increased KIR expression in T cells. KIR^+^ CD8 T cells efficiently eliminate gliadin-specific CD4 T cells from celiac disease patients. The frequency of KIR^+^ CD8 T cells is increased in virus-infected patients, including those affected by SARS-CoV-2 and influenza, and this increase in virions is associated with the severity of the disease. Notably, TCR sequencing analysis revealed that KIR^+^ CD8 T cells share a common TCR repertoire and antigen specificity that is independent of disease type^148^.

**Fig. 5:**
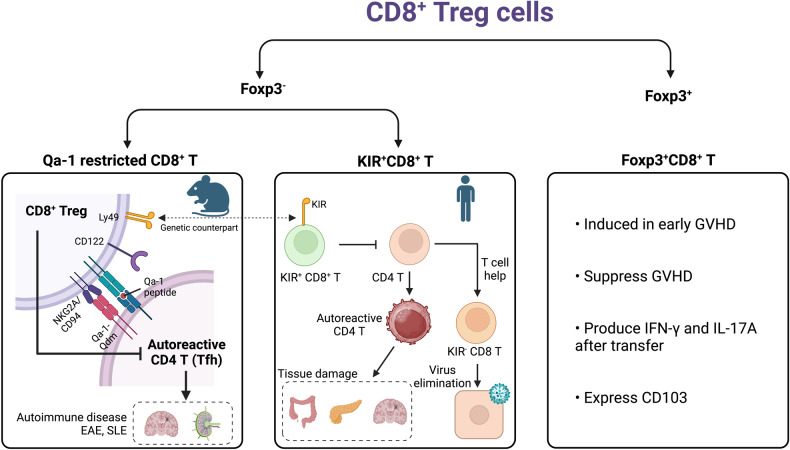
Types of suppressive CD8^+^ Tregs. Suppressive CD8^+^ Tregs are distinguished by the expression of Foxp3. Qa-1-restricted CD8^+^ T cells and KIR^+^ human CD8^+^ T cells do not express Foxp3. Qa-1-restricted CD8^+^ T cells recognize the Qa-1 peptide on MHC class I molecules of autoreactive Tfh cells and suppress autoimmune diseases. Suppressive human CD8 T cells express KIR, which is the genetic counterpart of the Ly49 molecule in mice, and suppress autoreactive CD4 T cells that can cause tissue damage in autoimmune disorders. Foxp3-expressing CD8 T cells are activated in an early GVHD model in vivo and can be converted into IFN-γ and IL-17A-producing CD8 T cells.

The other subset of regulatory CD8 T cells, known as Foxp3^+^CD8^+^ Tregs, can be induced during early graft-versus-host disease (GVHD). Through transcriptional profiling, it has been discovered that Foxp3^+^CD8^+^ Tregs exhibit a transcriptional signature consistent with canonical Foxp3^+^CD4^+^ Tregs and actively contribute to the suppression of GVHD by supporting Foxp3^+^CD4^+^ Treg function. Notably, in the absence of Foxp3^+^CD4^+^ Tregs, Foxp3^+^CD8^+^ Tregs efficiently prevent GVHD-induced severe inflammation, demonstrating their potent regulatory capabilities^150–152^. Surprisingly, 60% of adoptively transferred Foxp3^+^CD8^+^ Tregs produced IFN-γ, while 20% of these cells produced IL-17 in a GVHD model^152^. In vitro-differentiated CD103^+^Foxp3^+^CD8^+^ Tregs suppressed CD4 T-cell responses and ameliorated CD4 T-cell-mediated lupus nephritis by directly suppressing B-cell responses^153,154^. These findings collectively underscore the essential role of CD8^+^ Tregs in self-tolerance and highlight their potential significance in autoimmune disorders and viral infections such as SARS-CoV-2 (Fig. 5).

## Therapeutic potential of Tc subsets

Tc1 cells form the backbone of the cancer immunotherapies that have been applied successfully to date. More than seven ICI therapies^155–161^ and six CAR-T-cell therapies^162–167^ have been approved by the FDA for several types of malignancies. To improve the therapeutic outcome of cancer immunotherapy, combinations consisting of ICIs with other immuno-oncology agents have been evaluated^168^. Specifically, combining ICIs with an adenovirus-based tumor antigen vaccine, an IL-15 superagonist (N-803), an anti-OX40/4-1BB, and docetaxel has demonstrated therapeutic benefits in both hot and cold tumor models, synergistically triggering an immune response^169^. Furthermore, the success of CAR-T-cell therapy has encouraged researchers to develop similar treatments for diseases in addition to cancer^17^. Second-generation CARs targeting HBV-surface proteins S and L (HBV infection), fibroblast activation protein (fibrotic disease), CD19 or autoantigens (autoimmune diseases), or uPAR (senescence) have been invented, and clinical trials for these CAR-T-cell therapies are ongoing^16,17^.

Targeting Tc2 cells is a novel and appealing strategy for treating asthma patients. Specifically, a human sample study showed that TM30089 and montelukast, which block PGD2 receptors and leukotriene receptors of Tc2 cells, respectively, reduced IL-5/IL-13 production and attenuated the migration of Tc2 cells that had been induced by PGD2 and LTE4^58^. Clinically, fevipiprant, a PGD2 receptor 2 antagonist, demonstrated therapeutic advantages in specific subsets of asthma patients in Phase 2 trials^170,171^. Although Phase 3 studies failed to yield a statistically significant result, a subtle but consistent reduction in the rates of exacerbation was observed with increasing dosages of fevipiprant^172^. In addition, several studies showed that HIF-1α inhibitors reduced the hypoxia-enhanced differentiation of Tc2 cells and allergic responses in lungs^62,173^. Moreover, aminoglutethimide and vitamin D3 downregulated the enzymatic activity of CYP11A1, leading to reduced IL-4-mediated conversion of CD8 T cells to IL-13-secreting pathogenic CD8 T cells^61,174^. Adding vitamin D to a treatment regimen led to a statistically significant reduction in asthma exacerbation rates in adult asthma patients with low levels of vitamin D, although there has been no consistent evidence of this effect in the pediatric population^175^. Omalizumab, a recombinant anti-IgE monoclonal antibody that has demonstrated efficacy in asthma treatments^176^, has been found to reduce not only the IgE concentration but also significantly reduced the frequency of IL-13-secreting CD8 T cells in patients with allergic asthma^177^. In cases of dermatitis, blockade of histamine receptors using thioperamide or JNJ7777120 decreased hapten-induced local inflammation and IL-13 secretion by CD8 T cells, with a more pronounced inhibitory effect than that of CD4 T cells^69^. Oclacitinib, a Janus kinase inhibitor indicated for pruritus or atopic dermatitis in dogs^178^, has shown a significant reduction in Tc2 and Th2 cells, and this treatment effect was more pronounced for Tc2 cells than for Th2 cells^179^. Allergen immunotherapy, another potential strategy, has been shown to significantly reduce the intracellular expression of IL-4 by Tc2 cells in the context of intermittent allergic rhinitis^64^. A tendency toward reducing the ratio of Tc2 cells/Tc1 cells was observed, while no significant changes in the ratio of Th2 cells/Th1 cells was observed.

Tc9 cells show superior effector function for in the context of adoptive cancer immunotherapy. Compared to Tc1 cells, Tc9 cells showed less cytolytic activity in vitro but surprisingly elicited greater in vivo antitumor responses against advanced tumors in model mice^79^. In fact, TNF-α-induced tumor-specific Tc9 cells displayed enhanced antitumor capabilities compared to the those of control Tc9 cells. These enhanced abilities involved increased cell survival and proliferation mediated by STAT5 or nuclear factor-κB signaling^180^. Additionally, adoptive transfer of Tc9 and Th9 cells has shown led enhanced antileukemic activity compared with that of Tc1 cells while attenuating the severity of GVHD, thereby reinforcing the suggestion that Tc9 cells are attractive cancer immunotherapy targets^181^.

As Tc17 cells play crucial roles in the pathogenesis of autoimmune diseases and cancer, targeting Tc17 cells may be a promising therapeutic strategy. Ustekinumab, an IL-12/IL-23 inhibitor, led to a substantial and persistent reduction in peripheral blood Tc17 and Th17 cells, and this effect was accompanied by clinical improvement of cutaneous and mucosal lesions in Lichen Planus patients^182^. Furthermore, treatment with ursolic acid (UA), a small-molecule inhibitor of RORγt, has been shown to efficiently repress the exhaustion of CD8^+^ TILs and reduce the tumor burden in tumor-bearing mice^87^. In human metastatic melanoma cancer cells, UA exhibited potent antitumor effects^183^. A Phase I trial was conducted to assess the safety of multiple doses and evaluate the antitumor effect of UA^184^. Dimethyl fumarate (DMF), indicated for multiple sclerosis and psoriasis, has been observed to inhibit the production of IL-17 by Tc17 cells and thus alter the transcriptional profile, including increased IFN-γ secretion, to be similar to that of “CTL-like” cells^185^.

Tc22 cells play a role in eliminating cancer cells. Specifically, the administration of pantothenate, a CoA precursor known to enhance IL-22 production by Tc22 cells, in combination with immune checkpoint inhibitor (ICI) therapy has demonstrated promising outcomes by eliminating tumors in mice^186,187^. Although the adoptive transfer of both Tc1 and Tc22 cells exerts antitumor effects, on average, mice receiving Tc22 cells showed prolonged survival compared to that of mice that received Tc1 cells^103^. Thus, it might be prudent to polarize T cells into the Tc22 lineage when utilizing CAR- or TCR transduction-based T-cell immunotherapies.

Although the utilization of CD8^+^ Tregs in the clinic has not been realized thus far, mainly due to the challenges in their identification and characterization, there is growing interest in exploring various agents that can modulate CD8^+^ Tregs in the context of relevant diseases^188^. Blocking CD40/CD40L and ICOS/B7h led to potent induction of CD8^+^CD45RC^low/−^ Tregs and CD8^+^PD1^+^ Tregs, respectively, inducing tolerance in vivo in mice with GVHD^189,190^. Another study showed that anti‐CD45RC mAbs depleted CD45RC^hi^ naïve and effector memory T cells re-expressing CD45RA (TEMRA) but preserved the abundance CD45RC^lo^ Tregs, thereby inducing transplant tolerance efficiently in rats and humanized immune mice^191^. Interestingly, anti-CD3 mAbs have exhibited efficacy in promoting Foxp3^+^ CD8^+^ Treg activity and sustained amelioration of RA in mice and Type 1 diabetes mellitus in humans^192,193^. Additionally, IL-2 in combination with TGFβ as well as IL-34 alone have demonstrated the ability to expand both CD4^+^ Tregs and CD8^+^ Tregs, which may be leveraged to mitigate diseases such as lupus and GVHD^194,195^. Moreover, adoptive cell transfer of human CD8^+^ Tregs and their activation induced by TGF-β1 and rapamycin in mice with collagen-induced arthritis significantly reduced the levels of anti-collagen IgG antibody, clinical scores, and degree of cartilage destruction^153^. Additionally, human CD8^+^ CAR-Tregs have been shown to enhance the suppression of human skin rejection and GVHD in NSG mice^196^, suggesting a potential benefit of CD8^+^ CAR-Tregs in transplantation. Drugs that can potentially induce or block a certain Tc subset are summarized in Table 2. In summary, exploring the potential of Tc subsets holds great promise for the advancement of innovative therapeutics in human diseases.

**Table 2:** Drugs that potentially target certain Tc subsets.

	Drug	Disease (1st approval year)	Mode of action	Study on Tc subsets	Clinical status	Ref.
Tc1 cells	Pembrolizumab (Keytruda®)	Melanoma (2016), etc.	PD-1 inhibitor	Patient study	FDA approved	^155^
	Nivolumab (Opdivo®)	Melanoma (2014), etc.		Patient study	FDA approved	^156^
	Cemiplimab (LIBTAYO®)	cSCC (2018), etc.		Patient study	FDA approved	^157^
	Atezolizumab (Tecentriq®)	UC (2016), etc.	PD-L1 inhibitor	Patient study	FDA approved	^158^
	Durvalumab (Imfinzi®)	NSCLC (2017), etc.		Patient study	FDA approved	^159^
	Avelumab (Bavencio®)	MCC (2017), etc.		Patient study	FDA approved	^160^
	Ipilimumab (YERVOY®)	Melanoma (2010), etc.	CTLA-4 inhibitor	Patient study	FDA approved	^161^
	IDECABTAGENE vicleucel (Abecma®)	MM (2021)	CAR-T cells	Patient study	FDA approved	^162^
	Lisocabtagene maraleucel (BREYANZI®)	DLBCL (2021)		Patient study	FDA approved	^163^
	Ciltacabtagene autoleucel (CARVYKTI®)	MM (2022)		Patient study	FDA approved	^164^
	Tisagenlecleucel (Kymria®)	B-ALL (2017), etc.		Patient study	FDA approved	^165^
	Brexucabtagene autoleucel (TECARTUS®)	MCL (2020), etc.		Patient study	FDA approved	^166^
	Axicabtagene ciloleucel (YESCARTA®)	DLBCL (2017), etc.		Patient study	FDA approved	^167^
Tc2 cells	Fevipiprant	Asthma	PGD2 receptor 2 (DP2) inhibitor	Human study	Phase 3 failure	^59,172^
	TM30089			Human study	-	^58^
	Montelukast (Singulair®)	Asthma (1998), etc.	Leukotriene receptor inhibitor	Human study	FDA approved	^58,197^
	BAY87-2243, YC-1	Asthma	HIF-1α inhibitor	In vivo animal study	-	^62,173^
	Aminoglutethimide/Vitamin D3	Asthma	CYP11A1 inhibition	Ex vivo animal study	Clinical study	^61,174,175^
	Omalizumab (Xolair®)	Asthma (2003), etc.	IgE inhibitor	Patient study	FDA approved	^176,177^
	Thioperamide, JNJ7777120	Dermatitis	Histamine receptor inhibitor	In vivo animal study	Phase 2 failure	^69,198^
	Oclacitinib (Apoquel®)	Pruritus/AD in dogs (2013)	Janus Kinase Inhibitor	Ex vivo animal study	FDA approved	^178,179^
	Allergen immunotherapy	Allergic rhinitis (2014)	Tc2 cells	Patient study	FDA approved	^64^
Tc9 cells	Tc9 cells	Melanoma, GVL	Adoptive cell transfer	In vivo animal study	-	^79,180,181^
Tc17 cells	Ustekinumab (Stelara®)	Lichen Planus(Off-label used)	IL-12/IL-23 inhibitor	Patient study	FDA approved for psoriasis, etc.	^182,199^
	Ursolic acid	Melanoma	RORγt inhibitor	Ex vivo animal study	Ex vivo human/Phase 1	^87,183,184^
	Dimethyl fumarate (Tecfidera®)	MS (2013)	T-BET/STAT5 activation	Patients study	FDA approved	^185,200^
Tc22 cells	Pantothenate	COAD, PC	CoA activation	In vivo animal study	-	^186,187^
	Tc22 cells	Melanoma	Adoptive cell transfer	In vivo animal study		^103^
CD8^+^ Tregs	CD40Ig	GVHD	CD40/CD40L inhibition	In vivo animal study	-	^189^
	anti-ICOS mAb/CTLA4-Ig	GVHD	ICOS/B7h inhibitor	In vivo animal study	-	^190^
	Anti‐CD45RC mAb	GVHD	CD45RC^high^ cells depletion	In vivo animal study	-	^191^
	Anti-CD3 mAb	RA, T1DM	CD8^+^T cells activation	In vivo animal/patients study	-	^192,193^
	IL‐2/TGFβ	SLE	CD8+ Tregs	In vivo animal study	-	^194^
	IL‐34	GVHD	CD8+ Tregs	In vivo animal/human study	-	^195^
	CD8+Tregs	RA	Adoptive cell transfer	In vivo animal study	-	^153^
	A2-CAR CD8+ Tregs	GVHD	CAR-CD8+ Tregs	In vivo animal study	-	^196^

## Concluding remarks

The diverse subsets of CD8 T cells, including Tc1 cells, Tc2 cells, Tc9 cells, Tc17 cells, Tc22 cells, Tfcs, and suppressive CD8 Tregs, have emerged as critical players in immune responses and disease pathogenesis. These subsets exhibit distinct phenotypic and functional characteristics, enabling them to fulfill specific roles in various immune contexts.

Importantly, these different subsets of CD8 T cells have been investigated in a wide range of disease contexts, including cancer, autoimmune disorders, and viral infections. The abundances, functions, and interactions within the tumor microenvironment of the immune system have been associated with disease severity, treatment response, and patient prognosis. Therefore, these cells hold significant potential as biomarkers for disease prediction, prognosis, and therapeutic targeting.

The characterization of Tc subsets has greatly expanded our understanding of their intricate immune responses and roles in various diseases. Further research in these areas will continue to elucidate the complex interplay between CD8 T-cell subsets and their importance in health and disease, ultimately improving the quality of life and overall survival of individuals.
